# Outreach to Native Hawaiian and Pacific Islander communities to increase breast cancer screening and clinical trial awareness

**DOI:** 10.1186/s12885-026-16493-2

**Published:** 2026-07-08

**Authors:** Kirsten Chun, Mai-Phi Tran-Dang, Patricia Cornett, Cheyenne Tashombe, Micaela Kauhane, Abrar AL-Adhmi, Wendy Pavao, Christa Braun-Inglis, Erin Bantum, Jami Fukui

**Affiliations:** 1 University of Hawaiʻi John A. Burns School of Medicine, Honolulu, HI United States of America; 2 University of Hawaiʻi at Mānoa, Honolulu, HI United States of America; 3 University of Hawaiʻi Cancer Center, 701 Ilalo Street, Honolulu, HI 96813 United States of America; 4 The Queen’s Medical Center, Honolulu, HI United States of America

**Keywords:** Breast Cancer Screening, Clinical Trial Awareness, Community Health Educator, Outreach, Native Hawaiian, Pacific Islander

## Abstract

Racial and ethnic minority groups, including Native Hawaiian and Pacific Islander (NHPI) communities, experience multilevel barriers to cancer screening and enrollment into cancer clinical trials. Such barriers include low health literacy, mistrust in the healthcare system, and access to affordable healthcare. To address these barriers, a pilot outreach program was developed with the primary aims to build partnerships between rural Oahu communities and healthcare systems through collaboration between a Community Health Educator (CHE), a healthcare provider (HCP), and a clinic research coordinator (CRC); and identify and address barriers to breast cancer screening through a culturally appropriate, cost-effective approach, leveraging the CHE as the main intermediary to make real-time adjustments based on community needs.

In this pilot program, we utilized a culturally congruent and gender concordant Community Health Educator (CHE), medical oncology health care provider (HCP), and clinic research coordinator (CRC) team to engage with Oahu’s rural underserved communities to facilitate enrollment into the Tomosynthesis Mammographic Imaging Screening Trial (TMIST) breast cancer screening trial.

From June 2022 to June 2023, the CHE/HCP/CRC team facilitated 52 outreach events across Oahu that held approximately 20,000 attendees. A total of 92 women were screened for TMIST. Of those, 12 were enrolled in TMIST and an additional 32 participants received mammograms. The CHE/HCP/CRC team provided breast cancer education, connected participants to screening opportunities, and gathered feedback on perceived barriers. Insights from participants highlighted the critical role of the CHE in building trust, improving understanding of breast cancer screening, and fostering connections between the community and healthcare services.

This pilot program emphasizes the value of culturally tailored, community-based approaches in addressing screening disparities and strengthening partnerships in underserved populations.

## Background

Breast cancer screening and enrollment into clinical trials is limited among racial and ethnic minority groups due to various multilevel barriers. Only about 2–8% of eligible adult cancer patients enroll in a clinical trial [1]. Additionally, having low minority representation in clinical trials reduces the generalizability of findings and exacerbates outcome disparities [2–4]. 

Commonly identified barriers to breast cancer screening include low health literacy, mistrust in the healthcare system and poor access to healthcare. Low health literacy is associated with low mammography screening rates [5]. Medical mistrust and health information gaps are associated with low participation in breast cancer screening programs within vulnerable populations [6]. Other barriers contributing to the low breast cancer screening rates include, lack of reliable transportation, uninsured/underinsured and no onsite mammography available [6, 7]. 

Moreover, many of the studies that have identified these challenges focus on minority groups of Black and Hispanic populations, where studies that include Native Hawaiian/Pacific Islander (NHPI) communities are particularly scarce. Incidence and mortality from breast cancer among NHPI women are disproportionately higher than women of other races. From 2014 to 2018, Native Hawaiian females in Hawaii had the highest age-adjusted incidence and mortality rates of 169.5 and 24.9 respectively per 100,000 people [8]. These rates were statistically significant and exceeded the incidence and mortality rates of all other races such as Chinese, Filipino, Japanese, White, other Asians, and other races/ethnicities. Within the Micronesian Pacific Islander community, a prior study found that among 202 women aged 40 or older, eligible for mammography screening, 82% had never had a mammogram [9]. Based on self-reported assessments of barriers to mammography screening among racial and ethnic minorities, Asian/Pacific Islanders reported more psychological and knowledge-related barriers (e.g. fear or lack of knowledge) and cultural/immigration-related barriers (e.g. language) than other groups of women [10]. 

Minority communities have acknowledged the advantages of community outreach to address health disparities. Employing diverse strategies that include cultural and linguistic adaptations with patient navigators that prioritize building partnerships within the community to improve enrollment in clinical trials are particularly important [11–13]. 

There are established programs to address some challenges such as the breast and cervical cancer control screening program (BCCCP) [14] which provide insurance coverage for breast and cervical screening through Federally Qualified Health Centers (FQHC). The Hawai’i BCCCP was established, in partnership with the Center for Disease Control and Prevention, in 1993 to offer breast and cervical cancer screening services as part of the National Breast and Cervical Cancer Early Detection Program (NBCCEDP). The Hawai’i minority underserved NCI Community Oncology Research Program (HI MU NCORP) at the University of Hawaii Cancer Center, incorporates a community-guided intervention strategy for their clinical trials through their outreach and engagement office.

To bridge these healthcare systems/programs with the community, a Community Health Educator (CHE) offers a unique approach. Unlike many healthcare professionals who primarily work in a clinical setting, CHEs are embedded in the communities they serve. A CHE’s scope of practice includes conducting activities that promote local community education and outreach to underserved communities [15]. This includes delivering culturally tailored and evidence-based cancer education, engaging in outreach, identifying gaps to the integration of education and outreach efforts, and proposing strategies to resolve identified barriers. By incorporating a CHE, the timeliness of breast, cervical, and colorectal cancer screening and diagnosis can be improved among the underserved populations served by FQHCs [16, 17]. 

Our pilot program utilized a dynamic team to address the known barriers of breast cancer screening in NHPI communities. The team consisted of a CHE, medical oncology health care provider (HCP), and clinical research coordinator (CRC). The CHE/HCP team worked closely with the CRC to provide education on breast cancer, breast cancer screening, clinical trials and coordination to increase enrollment of the rural dwelling NHPI community to the Tomosynthesis Mammography Imaging Screening Trial (TMIST). The TMIST protocol is a nationally available IRB approved breast cancer screening trial, which allows for a wide outreach in the community. Other pilot projects using the CHE model in the PI community have been successful in accruing to the TMIST protocol to decrease screening disparities [17, 18]. 

This paper discusses our aims to: (1) build a partnership between the rural Oahu communities and healthcare systems via the CHE/HCP/CRC team and (2) identify and address barriers to breast cancer screening through a culturally appropriate and cost-effective approach, utilizing the CHE as the main intermediary, leveraging their ability to create real-time adjustments.

## Methods

A formal partnership between The Queen’s Medical Center (QMC), a BCCCP clinic, HI MU NCORP, a Federally Qualified Health Center (FQHC): Ko’olauloa Health Center, and a CHE/HCP/CRC team was initiated in January 2022.

Regularly scheduled weekly meetings with the CHE/HCP/CRC team helped to coordinate outreach events, breast cancer/clinical trial education, and facilitate breast cancer screening. From June 2022 to June 2023, the CHE/HCP/CRC team attended community, educational, and religious events to engage with rural Oahu’s underserved communities and facilitate enrollment into TMIST and increase breast cancer screening. Community outreach events included farmers markets and back-to-school events. Educational events included health fairs and healthcare provider community lectures. Faith based/religious events included women’s small group meetings and other church/spiritual gatherings.

At each outreach event, information on breast cancer screening was either given to attendees via direct communication by the CHE, UHCC brochures, questionnaires, and/or medical education lectures by the HCP. The CHE, a Pacific Islander woman, breast cancer survivor, and a patient advocate, who resides in rural Oahu, played an important role in these interactions; serving as the community event coordinator and lay cancer screening/clinical trial educator for TMIST and breast cancer screening participants. While the NHPI community is culturally and linguistically diverse, the CHE’s background allowed for meaningful points of connection. She was actively involved in Pacific Islander community networks including: Hawaii Alliance for Pacific Islanders, Native Hawaiian Pacific Islander Collaboration and Pathways for Advancement for Pacific Islanders. She is also fluent in Tongan, and frequently communicated with Tongan-speaking participants to overcome language barriers. Additionally, when sharing her breast cancer journey, the CHE utilized the “talk story” approach, which is a culturally rooted method of conversational communication that is common in the NHPI communities residing in Hawaii.

While engaging with the community at these events, the CHE/HCP team initiated the screening process for enrollment into TMIST by identifying potential participants to the CRC for further evaluation of eligibility. NHPI women residing in rural Oahu were approached for interest in TMIST trial and breast cancer screening. Eligibility for TMIST included: participants who were not receiving regular mammograms, women aged 45 to 69. Exclusion criteria for TMIST included: participants who had a mammogram within the last 11 months and/or had breast enhancements. The CRC coordinated with QMC/BCCCP to schedule participants for breast cancer screening, for which there were reserved time slots provided specifically for outreached participants. During each outreach event, the number of attendees and recorded demographics were quantified utilizing a hand counter and sign in sheet. The data collected originated from the CHE’s outreach efforts, and no individual consent was required from breast cancer screening participants, as the information was obtained through community engagement activities rather than direct patient research.

Adjustments to participant engagement were made in real time based on ongoing feedback the CHE received directly from the community members during events. Barriers and facilitators were identified through the CHE’s direct conversations with participants and through observations by the CHE/HCP/CRC team. These insights were discussed both during the events and at weekly scheduled meetings, where the CHE relayed participant perspectives and proposed modifications to the HCP/CRC healthcare team, which informed changes in how recruitment and education were carried out. One such refinement was the CHE’s development of educational sessions for the HCP/CRC staff, which enhanced cultural sensitivity and improved their ability to respectfully and competently engage with the community. Other changes to engagement practices, including use of plain language, holding smaller group discussions, and involving a senior CRC staff member, were introduced over time as the team learned from participant feedback. While not collected through formal surveys or structured interviews, these community-driven insights provided the foundation for the barrier/solution themes summarized in the Results.

Beyond the efforts of community education and outreach, the CHE’s responsibilities intersected with those of a patient navigator, such as providing individualized assistance to overcome barriers during the prediagnosis phase of cancer that interfere with compliance, such as mistrust in the health care system [19]. For example, the CHE personally provided transportation for participants interested in breast cancer screening. Another responsibility of the CHE to facilitate participants’ navigation in this was building rapport. The CHE would call participants before the mammogram as a proactive measure to identify potential psychological barriers [10], such as fear and anxiety, and provide tailored support early on. There were also additional opportunities immediately before and after the mammogram that allowed the CHE to directly engage with the participants to maintain rapport and a supportive environment.

## Results

**Fig. 1:**
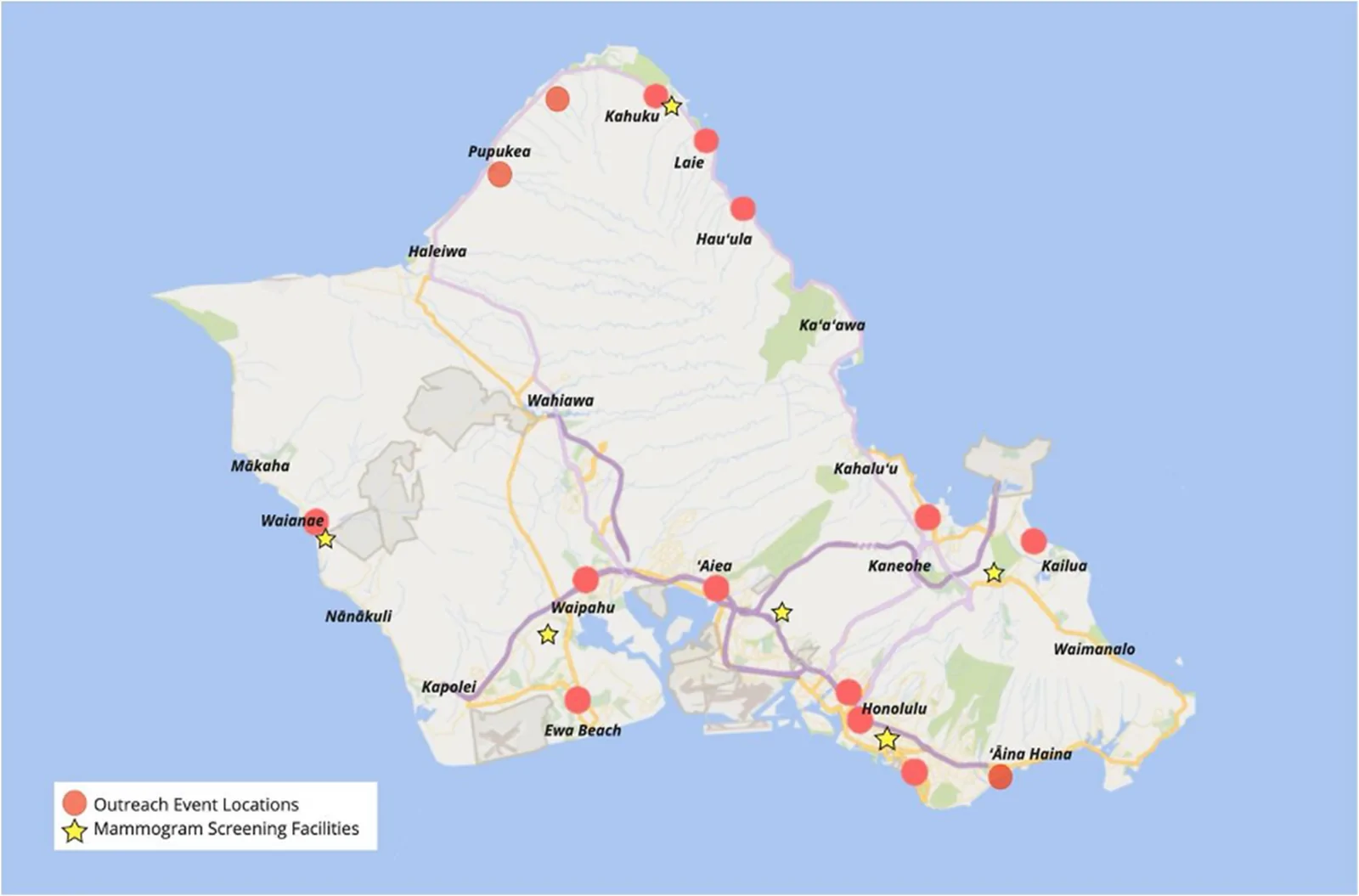
Distribution Map of Outreach Events and Mammogram Screening Facilities

From June 2022 to June 2023, there were 52 outreach events with an estimated 20,000 people in attendance. A majority of the outreach events were held at community gatherings (58%) followed by educational events (23%) and Religious events (19%). The largest estimated number of attendees were present at the community outreach events (82.7%) in comparison to Educational and Faith based (or Religious) events. Figure 1 illustrates the distribution of outreach events and location of mammogram screening facilities.

We reviewed participant intake forms to evaluate outreach efforts in NHPI communities in the rural areas of Oahu. Participant demographics were categorized by (1) Age, (2), Ethnicity, (3) Citizenship, and (4) Insurance. The participant demographics are listed in Table 1.

A total of 92 women participated in the screening process for the TMIST clinical trial. Participants ranged in age from 24 to 85 and were mostly Pacific Islander (42%) and Native Hawaiian/Mixed Native Hawaiian (36%). Other ethnicities of participants included White, Hispanic, Asian and Mixed-ethnicities not including NHPI. The majority of participants had citizenship status (80%) and private insurance (65%).

**Table 1:** Demographic Table of Outreached Participants from 2022–2023

Variables	Screened Participants* (*N* = 92), *N* (%)
Age group, years	
20–29	7 (7.6)
30–39	7 (7.6)
40–49	21 (22.8)
50–59	38 (41.3)
60–69	11 (12.0)
70–79	7 (7.6)
80+	1 (1.1)
Ethnicity	
White	11 (11.9)
Hispanic	2 (2.2)
Asian	6 (6.5)
Native Hawaiian	16 (17.4)
Pacific Islander	39 (42.4)
Mixed - NHPI	17 (18.5)
Mixed - Other	1 (1.1)
Citizenship	
Yes	74 (80.4)
No	18 (19.6)
Insurance	
Private	60 (65.2)
Medicare	3 (3.3)
Medicaid	19 (20.7)
Not Disclosed	10 (10.9)

*8 participants completed annual mammogram prior to outreach

Of the 92 participants who went through the screening process, 12 were ultimately enrolled in TMIST and an additional 32 participants received mammograms (Non-TMIST group). The remaining 48 participants were ineligible for TMIST and mammogram screening or not interested in participating in the clinical trial or receiving their annual mammogram.

Through the “talk story” approach, the CHE built rapport, normalized discussion on cancer, and created a safe space of open dialogue, which facilitated further exploration of participants’ prior beliefs. Having access to this perspective permitted the CHE to challenge participants’ resistant beliefs to cancer screening.

The real-time adjustments made by the CHE helped improve communication and strengthen relationships between the HCP/CRC team and underserved communities. For example, the CHE brought attention to the negative connotation of the word “cancer” and explained the discomfort participants felt within a large group setting. Therefore, adjustments were made to not obviously display the UHCC logo at events and focus on smaller group meetings for cancer screening/clinical trial education with the hope of forming a better connection with the community and participants.

During the enrollment process of the TMIST study, the CHE shared that fear and discomfort were commonly experienced by participants when medical terminology was used in explaining the TMIST trial. Based on this feedback, the CRC staff utilized plain language to improve cultural competency [20] and address the low health literacy aspects encountered during the clinical trial screening process. In addition, the CHE explained to younger CRC staff the significance of having an older woman conduct the screening for the trial to garner respect and foster a sense of trust for participants during the appointments.

Through community outreach with direct CHE engagement and development of the CHE/HCP/CRC team, we determined several key barriers to breast cancer screening and TMIST clinical trial enrollment in the NHPI community. These barriers were addressed in real-time and are summarized below. (1) Lack of perceived importance, (2) Medical mistrust, and (3) Transportation concerns or lack of access to health centers (Table 2).

Table 2 shows examples of participant discourse obtained through direct communication with the CHE and how the CHE responded to these challenges in real-time. Participants frequently voiced concerns that research data would not benefit their community, noting past experiences where data were collected from NHPI populations without visible impact. To address this mistrust, the CHE emphasized how TMIST findings could inform breast cancer screening guidelines relevant to NHPI women and assured participants that results would be shared back with the community. In addition, the CHE/HCP/CRC team adapted screening practices, such as implementing a senior female CRC to conduct TMIST appointments to foster cultural respect and increase participant comfort.

**Table 2:** Barriers to Participant Engagement in Breast Cancer Screening and TMIST Trial

Barrier	Discourse Examples	Solutions Implemented
Low health literacy	“…treated as a guinea pig” specifically when discussing verbiage that was intimidating or confusing (i.e. clinical trial protocol consent language) “Surveys don’t really count for anything.”	Utilized plain lay language to discuss trial concepts “Talk story” with participants and explain significance of breast cancer screening
Medical mistrust	“Why am I doing this survey? Is it so you can be paid?” “Is this data going to help my people?”	Direct communication through smaller groups Host education sessions on cultural sensitivity and awareness for CRC and HCP Remove the large UHCC “cancer” logo banner at events CHE shared her story and the importance of early detection with mammography Ensured a senior female CRC conduct TMIST screening to foster cultural respect and trust
Transportation concerns Insurance coverage	30 miles is the average distance to the TMIST mammogram screening center, QMC Mammogram copayments required.	Offer and provide transportation to all interested participants Utilize established BCCCP clinics and other cost support available at FQHC

## Discussion

Prior to this pilot project, there were little to no formal partnerships set in place that utilized a CHE/HCP/CRC team to gain insight into barriers that NHPI communities in rural Oahu were experiencing and to address these barriers, with the goal to increase cancer screening and clinical trial participation. Through the efforts of the CHE/HCP/CRC team, this pilot project helps address multiple identified barriers using an innovative and collaborative approach to sustain a community-facing partnership utilizing the clinical trial infrastructure from TMIST and whose strategy can be adopted to include other important clinical trials to reduce health disparities. Fostering these collaborations is a potentially powerful tool to achieve public health goals.

We systematically incorporated the data/insights gained from CHE/HCP/CRC into cancer screening/clinical trial processes. Since direct feedback from the communities is paramount and can be facilitated through a CHE, having a CHE/HCP/CRC team is critical to make real-time improvements to the process while promoting patient engagement and access to care [21]. Through the efforts of the CHE/HCP/CRC team, we identified several social determinants of health for the NHPI populations in Hawaii, which will be discussed below.

### Low health literacy and medical mistrust

Low participation in cancer screening is associated with low health literacy and medical mistrust, in which the mistrust is often grounded in historical and ongoing systemic inequities. Examples of historical inequalities include the unethical sterilization of Indigenous women, illustrating how marginalized communities’ hesitancy toward healthcare institutions stems from lived experience and documented harm [22]. Similar experiences within the NHPI and other underserved populations, along with ongoing disparities in care, may contribute to reluctance toward breast cancer screening. These experiences compounded with misguided or incomplete information about breast cancer and its prevention can further hinder participant’s informed decision-making about mammography screenings. Addressing these barriers involves the contribution from a committed healthcare team, starting with the healthcare provider. Healthcare providers immersed in the community they serve results in direct and sustained connection with their patient population and establishes trust. Being engaged in the needs of the community prepares the healthcare team for patient resistance to screening and helps to address social determinants of health and care [23]. In this pilot project, the CHE was able to (1) gently challenge participants’ attitudes towards breast cancer screening and (2) elucidate major burdens, such as transportation and time off work, by being involved in the community. Greater understanding of patient-specific barriers within diverse populations could help healthcare providers connect to local resources to support breast cancer screening.

Community-based outreach navigation programs also continue to be used as a resource to increase screening in the community [24]. Demonstrating cultural sensitivity while sharing cancer screening education helped build rapport and was essential for a positive experience and reception of sensitive information. Our CHE, who is from the community and has a personal experience with the cancer screening process and cancer journey was crucial in fostering trust and encouraging participation in screening efforts. The way she shared her personal experiences with participants through “talk story” was central to her ability to connect. Rather than direct delivery of information, this conversational exchange facilitated trust-building, normalized discussions around cancer, and revealed barriers that might not have been identified through formal education alone. This process reflects the dialogue-based approach of lay health worker engagement described in other community health interventions [25]. 

Having a CHE with a personal connection to the community with a shared understanding of cultural nuances may allow for deeper, more meaningful interactions and encourage receptiveness. This approach established relatable outreach and helped the community feel engaged in supporting the cancer screening process.

### Access to healthcare

Administering successful cancer screening depends on social context [26]. The lack of awareness of study protocols and the perceived cost burden on patients, are significant contributors of healthcare providers inability to enroll patients into clinical trials [27]. Together with the CHE, the CRC team was able to assess accessibility for uninsured and underinsured patients for breast cancer screening during the TMIST clinical trial eligibility screening period. This also allowed the CRC team to learn about specific needs and ways to accommodate participant preferences-mammogram location, co-pays for mammography, which remain a major challenge in cancer screening. Selecting trials that are designed to better match population needs, minimizes participant burdens during recruitment and enhances trust and engagement [20]. Trials could be designed to assist participants in overcoming barriers, such as covering costs that impose hurdles to participation [28]. In the beginning stages of the program, it became evident that mammogram co-pays were a challenge for some participants. We explored several potential funding sources but were not able to routinely offset these costs. This highlights the implicit bias of a study to assume the screening mammography costs to potential participants would be acceptable. Unfortunately, this aspect was particularly burdensome for underinsured participants and shows the importance of recognizing these inherent barriers to care. We have since established a Pacific Island Community Advisory Board that is composed of respected community leaders, healthcare professionals, and cultural experts, in which they oversee research projects serving the Pacific Islander community. The barriers in research study design could potentially be addressed in real-time through these groups, leading to increased inclusion of participants in these communities.

Transportation difficulties have consistently been one of the most significant barriers to cancer screening [6]. Public transportation options are limited in rural NE Oahu, requiring patients to rely on family and/or friends for transportation and/or coordinate costly arrangements like taxis, which can lead to scheduling conflicts and delays in getting care/screened. In response, the CHE directly addressed this barrier by providing transportation for all interested participants. Having a CHE from rural communities who understands the challenges of limited access would be invaluable if implemented across all rural areas on Oahu and neighboring islands.

Attention should be given to care facility location and care provision [23]. Transportation is essential to consider because it is a logistical obstacle that could hinder participants from receiving better resources for breast cancer screening. In this outreach program, many of the participants receiving mammograms preferred to be transported into the urban city. Although these health care facilities were further, the urban health care facility-QMC a BCCCP clinic, provided more support and resources, including access to the national breast cancer screening clinical trial. By addressing transportation barriers, participants have improved access to resources for cancer screening, enhancing the overall quality of care.

Duplicative efforts can be another burden to healthcare providers, especially between primary care and oncology providers. Since the primary care facilities did not have an established breast cancer screening program, our pilot program augmented primary care efforts for cancer screening. As complementary efforts, the CHE/HCP/CRC team connected with primary care clinics at FQHCs in rural Oahu to develop a pathway for patients to undergo screening regardless of eligibility or participation in the TMIST study. Establishing communication with participants’ primary care providers was essential to ensure a smooth follow-up transition and ensure no gap in care.

### Limitations

Our project included a diverse population with unique cultural aspects. The tailored strategies used here to address barriers to clinical trial engagement may not be applicable to other programs and populations as prior studies show heterogeneity between patient-navigation/outreach program outcomes causes difficulty in evaluating across different programs and populations [29]. However, the common lessons learned to address the barriers that were identified in our project could still be applied. In addition, our project heavily relied on our CHE, which might not be available routinely in some communities.

## Conclusion

Our NHPI communities continue to have high incidence and mortality rates from breast cancer with low participation in both breast cancer screening and clinical trials. Although there are many studies that identify and describe barriers to patient enrollment and proposed interventions to address such challenges, there are few studies that focus on the NHPI population and implement the proposed interventions to address identified barriers in real time. We built a formal partnership between HI MU NCORP, QMC (TMIST site and a BCCCP clinic), a rural FQHC (without a formal cancer screening program), and a CHE/HCP/CRC team to increase accrual to TMIST by enhancing an existing strategy [18]. As the partnership continues to mature, we hope this project contributes towards equity in communities with low breast cancer screening and clinical trial participation rates as well as uncovers new barriers and solutions to those barriers through participant engagement.

## Data Availability

No datasets were generated or analysed during the current study.
